# Supplementary material for: Content-rich biological network constructed by mining PubMed abstracts
Source: BMC Bioinformatics. 2004 Oct 8;5:147. doi: 10.1186/1471-2105-5-147 (PMC528731; doi:10.1186/1471-2105-5-147)
Supplement: Additional File 2 — The original results of the above study (non-essential files are deleted to keep the file size under the limit set by BMC bioinformatics). [file 1471-2105-5-147-S2.bz2 › chilibotAdditionalFile2/dip05/234ID8006584E758/html/IL2RB_IL2RA.html]

 


 **IL2RB** and **IL2RA** 
  
Found 1183 abstracts in PubMed, retrieved 05.  
 

 What does Google say? 
 PDF only 
| .edu only 

---

**Interactive relationship** (e.g. stimulation, inhibition, etc)

**Non-interactive relationship** (e.g. studied together, co-existance, homology, etc.)

- Most investigated Treg cells are CD4 pos cells expressing the alpha chain of the IL 2 receptor  [ **IL2RB** ]  CD25  [ **IL2RA** ] .  Ref: 12848995 Autoimmun Rev, 2002
- Activation markers CD25  [ **IL2RA** ] , CD122  [ **IL2RB** ] , CD69 and CD80 were also present on fetal T cells.  Ref: 12880639 Dev Comp Immunol, 2003
- The inhibitory effect on human T cells was assessed and demonstrated at the level of proliferation DNA synthesis, IL 2 secretion, and IL 2 receptor  [ **IL2RB** ]  CD25  [ **IL2RA** ]  upregulation.  Ref: 12835898 Inflamm Res, 2003
- We found a defective T cell response in malnourished children, as indicated by diminished levels of circulating total CD3, helper CD4, IL 2 receptor  [ **IL2RB** ]  bearing CD4 CD25  [ **IL2RA** ]  and memory helper T cell responses CD4 CD45RO in keeping with the decreased specific IgE levels against Ascaris lumbricoides.  Ref: 12848201 J Trop Pediatr, 2003
